# Development of a Longitudinal Research Curriculum for Pediatric Emergency Medicine Fellowship

**DOI:** 10.5811/westjem.2021.10.52854

**Published:** 2022-01-01

**Authors:** Ankita Taneja, Todd Wylie, Colleen Kalynych, Haytham Helmi, Jennifer Fishe

**Affiliations:** * University of Maryland School of Medicine, Department of Emergency Medicine, Baltimore, Maryland; † University of Florida College of Medicine – Jacksonville, Department of Emergency Medicine, Division of Pediatric Emergency Medicine, Jacksonville, Florida; ‡ University of Florida College of Medicine – Jacksonville, Office of Educational Affairs, Jacksonville, Florida; § University of Florida College of Medicine – Jacksonville, Department of Emergency Medicine, Jacksonville, Florida

## BACKGROUND

The Accreditation Council for Graduate Medical Education (ACGME) requires programs to develop research curricula regarding how research is “conducted, evaluated, explained to patients, and applied to patient care.”^1^ Specific to fellowship, the ACGME requires fellows to participate in and complete scholarly work aligned with their subspecialty requirements.^2^ The American Board of Pediatrics (ABP) subspecialty in pediatric emergency medicine (PEM) further requires that each PEM fellow have a strong core knowledge in scholarly activities and complete meaningful scholarly work; the ABP tests research knowledge as part of the in-training exam (ITE), board certification exam, and maintenance of certification. The ITE and board exams’ proportion of research questions is not trivial (7% of questions overall).^2^,^3^

However, how to optimally merge research education with individual fellowship research projects is unknown. Further to this, individual trainees’ level of research education varies widely.^4^ For fellowship where research is of higher emphasis, traditional research “blocks” are not well suited to completing a substantive project given unpredictable and variable time periods to obtain institutional review board (IRB) approval and acquire/analyze data. Therefore, fellows need a structured research curriculum to perform well on the ITE and board exams and finish their research project, 5,6 while also fulfilling clinical duties and maintaining wellness. Additionally, a MedEdPortal search at the commencement of this project revealed no PEM fellowship research curricula. At our institution, the previous approach for addressing those areas was a monthly PEM journal club, individual dedicated research blocks, and the completion of a scholarly project by each fellow. However, a program needs assessment revealed that scholarly activity questions on the ITE exam was the section where fellows scored lowest.

## OBJECTIVES

We developed a longitudinal research curriculum for PEM fellows using Kern’s six-step approach. Our goals were to 1) impart research knowledge; 2) ensure completion of individual research projects; and 3) adequately prepare fellows for board exams. We present preliminary data regarding the third goal using fellow ITE scores before and after the curriculum’s implementation.

## CURRICULAR DESIGN

Our ACGME-accredited PEM fellowship contains seven fellows in a three-year program, housed within the emergency department of a large academic hospital/university. The previous curriculum used fixed research “blocks.” We implemented the new research curriculum at the beginning of academic year 2018–2019. This educational initiative was exempt from IRB approval. We used Kern’s six-step approach to assist in developing a longitudinal curriculum (Table 1).^4^,^7^ As stated above, we recognized the general need by reviewing previous fellow research performance on the ITE exam. We assembled a multidisciplinary group comprised of the PEM fellowship director, a second-year PEM fellow, the PEM fellowship’s new research rotation director, and our campus-wide Office of Educational Affairs’ Director of Educational Development and Research to complete a targeted needs assessment. Decisions were made by consensus of individuals’ opinions related to their subject matter expertise using a Robert’s Rules of Order approach.

The group conducted a search for disseminated/published research curricula and reviewed the ABP’s four core-knowledge content areas. Building upon those four areas, we structured six key topic areas to follow a longitudinal semester-type format over the three years of fellowship: Ethics in Research; Principles of Epidemiology and Clinical Research Design; Principles of Biostatistics in Research; Statistical Testing; Measurement of Association and Effect; and Cost Benefit, Cost Effectiveness, and Outcomes. We then developed learning objectives within each of the topic areas (Table 1). To provide content for each of those objectives, we performed a search of formal gray literature, followed by vetting selected online learning modules, videos, and written materials that could serve as independent learning content. We used the online learning management system Canvas (Instructure Inc., Salt Lake City, UT) to organize the materials and for easy access for the fellows. Additionally, each semester contained milestones for fellows to complete work toward their scholarly projects, and those milestones were tied to the other educational research content. The PEM fellowship’s research rotation director formatted the monthly literature review to tie articles to key semester content.

## IMPACT/EFFECTIVENESS

This project produced a longitudinal structured research curriculum for pediatric EM. Our preliminary evaluations include fellow reviews and ITE scores. Fellow reviews of the new curriculum were universally positive, particularly commenting on the benefit of greater time to develop and complete their research projects, including comments such as “have enough time getting my research project done,” “EXCELLENT” resources and materials, and “exposure to research, time to work on research project.” We compared de-identified fellows’ ITE scores from the year before and after curriculum implementation, specifically questions on core knowledge in research. Overall, the seven fellows’ median score rose from 37.5% to 75% correct (score range pre-curriculum 25–75% and post-curriculum 37.5–87.% correct). Small sample size precluded further statistical testing for this preliminary, short-term data of effectiveness.

We also believe the core content of research curriculum is transferrable to other fellowship programs regardless of specialty. A major advantage of our curriculum is its longitudinal structure, which accounts for and is resilient to the unpredictable vagaries in completing scholarly projects (eg, waiting for IRB approval, data analysis, etc.). Longitudinal curricula have been shown to provide learners with an innovative, practical educational vehicle to achieve their educational goals.^8^ Longitudinal curricula also emphasize patient- and learner-centered education and are better suited to prepare trainees for lifelong learning.^9^,^10^

This curriculum was also strengthened by its multidisciplinary inception and active involvement of a PEM fellow in its construction. We feel the multidisciplinary approach and trainee involvement contributed to the semester structure and balance of topics toward better feasibility and sustainability, although more longitudinal follow-up is required to draw conclusions in that regard. Our curriculum was also designed to follow the natural progress of a scholarly project over the three years, as the first modules focus on ethical research conduct, epidemiology principles, and study design to aid in formulating a hypothesis. This was followed by biostatistics content for the third and fourth semesters when fellows would be expected to engage in data analysis for their individual projects. Additionally, given that there is a plethora of open-source online educational material on this topic, we found the program relatively easy to implement and at minimal cost.

## LIMITATIONS

Our curriculum and this project have limitations. The improvement in ITE scores is over one year and needs further monitoring as more fellows complete and progress through the program. Differences pre- and post-curriculum could be due to fellowship level, more experience with scholarship, or other non-curriculum related gains in knowledge. While ITE scores in some cases correlate with future board exam performance, that finding too requires future evaluation.^11^ Future evaluations of the curriculum should include the quality of fellows’ individual research projects, including conference presentations and peer-reviewed publications. The curriculum is designed to meet the ABP’s content specifications for PEM, and while very broad and likely generalizable to other specialties/subspecialties, may not be completely transferrable. Lastly, we tested the curriculum at one program; thus, more generalizable evidence from other institutions is needed.

## CONCLUSION

We constructed and implemented a longitudinal PEM fellowship research curriculum. Future work remains to measure our curriculum’s impact on individual fellow research projects, long-term board exam performance, and adaptability to other institutions and programs.

## Figures and Tables

**Table 1:** Pediatric emergency medicine research curriculum learning objectives and milestones by postgraduate year.

Semester	Learning objectives	Learning content	Milestones for scholarly project
Semester 1		Ethics in research	Institutional IRB training
July – Dec PGY 4	Understand the principles of ethical conduct of human subjects research Acquire a familiarity with univariate statistical testing techniques	1. Professionalism and misconduct in research 2. Principles of research with human subjects 3. Principles of consent and assent 4. Diagnostic tests 5. Including “gold standard” in testing, sensitivity, specificity	Identify mentor / topic / hypothesis
Semester 2		Principles of Epidemiology and Clinical Research Design	IRB approval for project
Jan – June PGY 4	Understand the importance of study design and pros and cons to different types of study designs	1.Assessment of study design, performance and analysis (internal validity) 2. Assessment of generalizability (external validity) 3.Bias and confounding 4. Causation a. Understand the difference between association and causation 5. Incidence and prevalence a. Distinguish disease incidence from disease prevalence	Introduction to manuscript written and approved by mentors and research rotation director
Semester 3		Principles of Use of Biostatistics in Research	Data abstraction completed
July – Dec PGY 5	Learn to formulate a proper hypothesis Understand and recognize different types of common data distributions	1. Types of variables 2. Distribution of data 3. Hypothesis testing	
Semester 4		Statistical Tests	Data analysis completed
Jan – June PGY 5	Apply knowledge learned to date to interpretation of results for scholarly research project		Submission of abstract to national conference
Semester 5		Measurement of Association and Effect	
July – Dec PGY 6	Understand the rationale for and be able to interpret results of advanced statistical tests including multivariate tests	1. Relative risk, risk ratio, odds ratio 2. Regression analysis	Initial draft of entire scholarly manuscript due to research rotation director
Semester 6		Cost Benefit, Cost Effectiveness, and Outcomes	Submission of scholarly manuscript for publication in a peer-reviewed journal
Jan–June PGY6	Become familiar with the vocabulary and variables used for economic evaluation of health services and outcomes		

*PGY*, postgraduate year; *IRB*, institutional review board.
